# Evolution and Adaptation of the Mitochondrial Protein Import Machinery in Parasitic Protists: Beyond the Canonical TOM Complex

**DOI:** 10.3390/ijms27188205

**Published:** 2026-09-15

**Authors:** Enayat Anvari, Gholamreza Daryabor, Amin Sedigh, Sajad Rashidi, Paul Nguewa

**Affiliations:** 1 Department of Physiology, School of Medicine, Ilam University of Medical Science, Ilam, Iran; 2 Autoimmune Diseases Research Center, Shiraz University of Medical Sciences, Shiraz, Iran; 3 Molecular Medicine Research Center, Khomein University of Medical Sciences, Khomein, Iran; 4 Department of Basic Sciences, Khomein University of Medical Sciences, Khomein, Iran; 5 Areas of Microbiology and Parasitology, Department of Molecular Biology, University of Navarra, IdiSNA (Navarra Institute for Health Research), c/Irunlarrea 1, 31008 Pamplona, Spain

**Keywords:** TOM complex, mitochondrial protein import, parasitic protists, mitochondrion-related organelles, host–pathogen interactions, ATOM complex, drug target

## Abstract

The translocase of the outer membrane (TOM) complex is the primary entry gate for nucleus-encoded mitochondrial proteins and is conserved across eukaryotes; however, parasitic protists exhibit remarkable adaptations. This review synthesizes current knowledge on the composition, structure, and function of TOM and related import systems in apicomplexans, kinetoplastids, and anaerobic protists with reduced mitochondrion-related organelles (MROs). We highlight major evolutionary innovations, including the bacterial-origin ATOM complex in trypanosomes—comprising core channel ATOM40, receptors ATOM46/69, structural subunit ATOM19, and multifunctional pATOM36 linking protein import to mitochondrial DNA inheritance—alongside lineage-specific receptors such as Tom60 in *Entamoeba histolytica* and extreme reductive streamlining in microsporidian mitosomes. Beyond its canonical role in biogenesis, we explore how parasites co-opt the host TOM complex for intracellular survival and how the import machinery integrates with broader cellular processes, including immune signaling, stress responses, and organelle dynamics. Notably, the essential nature of these adapted systems, coupled with their significant divergence from host machinery, highlights their potential as targets for developing novel therapeutic strategies against major parasitic diseases. This work underscores that understanding the diversity of mitochondrial protein import pathways not only provides insights into eukaryotic evolution but may also open new avenues for the development of antiparasitic drugs.

## 1. Introduction

Mitochondria are essential organelles in eukaryotic cells, responsible for vital processes such as energy production, iron–sulfur cluster assembly, and the regulation of apoptosis. The vast majority of mitochondrial proteins (approximately 99%) are encoded by nuclear genes, synthesized in the cytosol, and subsequently imported into the organelle through highly specialized protein translocases. The translocase of the outer membrane (TOM) complex forms the obligate entry gate for virtually all mitochondrial precursor proteins, serving as the initial recognition and translocation point for proteins destined to all mitochondrial subcompartments [1,2].

The TOM complex is a multi-subunit molecular machine that is remarkably well-conserved from yeast to humans [1,2,3,4]. However, recent investigations into diverse parasitic protists have revealed that this canonical arrangement represents only one feature of the mitochondrial import machinery. Parasites often inhabit specialized niches and exhibit outstanding adaptations in their metabolic pathways and organelles, particularly their mitochondria [5,6]. These adaptations are reflected in the protein import machinery, with many parasites possessing highly modified, reduced, or entirely divergent TOM complexes [7,8]. For instance, trypanosomatids have replaced the canonical TOM complex with an entirely different complex termed archaic translocase of the outer mitochondrial membrane (ATOM) [7], while anaerobic parasites with reduced mitochondrion-related organelles (MROs) such as mitosomes and hydrogenosomes often retain only a minimal core import machinery [9]. Notably, the central pore of this complex, ATOM40, is not a divergent Tom40 but is of direct bacterial origin, belonging to the outer membrane protein 85 (Omp85) superfamily [10]. Furthermore, components like protein associated with the ATOM complex, 36 kDa (pATOM36), have evolved multifunctional roles, directly coupling the essential process of protein import to the unique mechanism of mitochondrial DNA inheritance in these parasites [11].

Ultimately, this review synthesizes current knowledge to offer a unified perspective on the remarkable evolutionary plasticity of the mitochondrial protein import machinery. We critically evaluate how adaptations in parasitic protists extend beyond reductive simplification to represent active, sophisticated innovations that facilitate survival within specific host environments. By systematically comparing these divergent systems—from streamlined apicomplexan complexes to the reinvented ATOM complex of trypanosomes—we establish the parasite-adapted import apparatus as a cornerstone for understanding mitochondrial biology in the context of infectious diseases, while highlighting these unique solutions as a rich source of novel principles in eukaryotic cell evolution.

## 2. The Canonical TOM Complex: Structure and Core Function

### 2.1. Basic Composition and Structure

The TOM complex is a multi-subunit molecular machine that forms the primary entry gate for proteins into mitochondria. The central pore is formed by Tom40, a β-barrel protein that serves as the essential channel for protein translocation. This core component is supported by three small regulatory subunits—Tom5, Tom6, and Tom7—which modulate the stability and dynamics of the complex. Recent high-resolution structural studies have revealed that the core complex functions as a dimer, with two Tom40 pores each associated with their complement of small Tom proteins [1,12].

This dimeric architecture is bridged by two molecules of Tom22, a central receptor that plays multiple critical roles. Tom22 possesses a cytosolic domain that functions as a docking site for the peripheral receptors Tom20 and Tom70, and an intermembrane space domain that facilitates the handover of translocating precursors to downstream translocases, such as the TIM23 complex [1]. The stability of this complex architecture is differentially influenced by its small subunits; Tom6 plays a major role in TOM complex stability, whereas Tom7 has a lesser but still significant function. Consistently, the genetic inhibition of both Tom6 and Tom7 results in a pronounced destabilization of the TOM complex, which induces severe growth defects, underscoring its essential cellular role [12,13].

### 2.2. Receptor Functions and Substrate Recognition

The import pathway is initiated by peripheral receptors that confer substrate specificity to the complex. Tom20 acts as the primary receptor for the majority of precursor proteins targeted to mitochondria by N-terminal presequences, which form amphipathic α-helices that dock into its hydrophobic cleft [14,15]. In contrast, Tom70 is specialized for recognizing hydrophobic precursors with internal targeting signals, such as metabolite carriers [15,16].

Tom70 facilitates the import of these hydrophobic precursors through dynamic interactions with cytosolic chaperones Hsp70 and Hsp90. Both Tom20 and Tom70 are structurally anchored to the core complex via Tom22, whose cytosolic domain acts as a central docking site, creating a unified gate where diverse protein substrates converge for translocation through the Tom40 channel [15].

## 3. Reductive Evolution in Anaerobic Parasitic Protists

Anaerobic parasitic protists, including *Giardia intestinalis*, *Entamoeba histolytica*, and *Trichomonas vaginalis*, possess highly reduced mitochondrion-related organelles (MROs) such as mitosomes and hydrogenosomes. These organelles have undergone extensive reductive evolution, losing many typical mitochondrial functions while retaining essential pathways such as iron–sulfur cluster assembly [9,17,18,19]. This reductive process is reflected in their protein import machinery, which often exhibits extreme streamlining while maintaining a core import function.

### 3.1. E. histolytica and the Novel Tom60 Receptor

**Figure 1:**
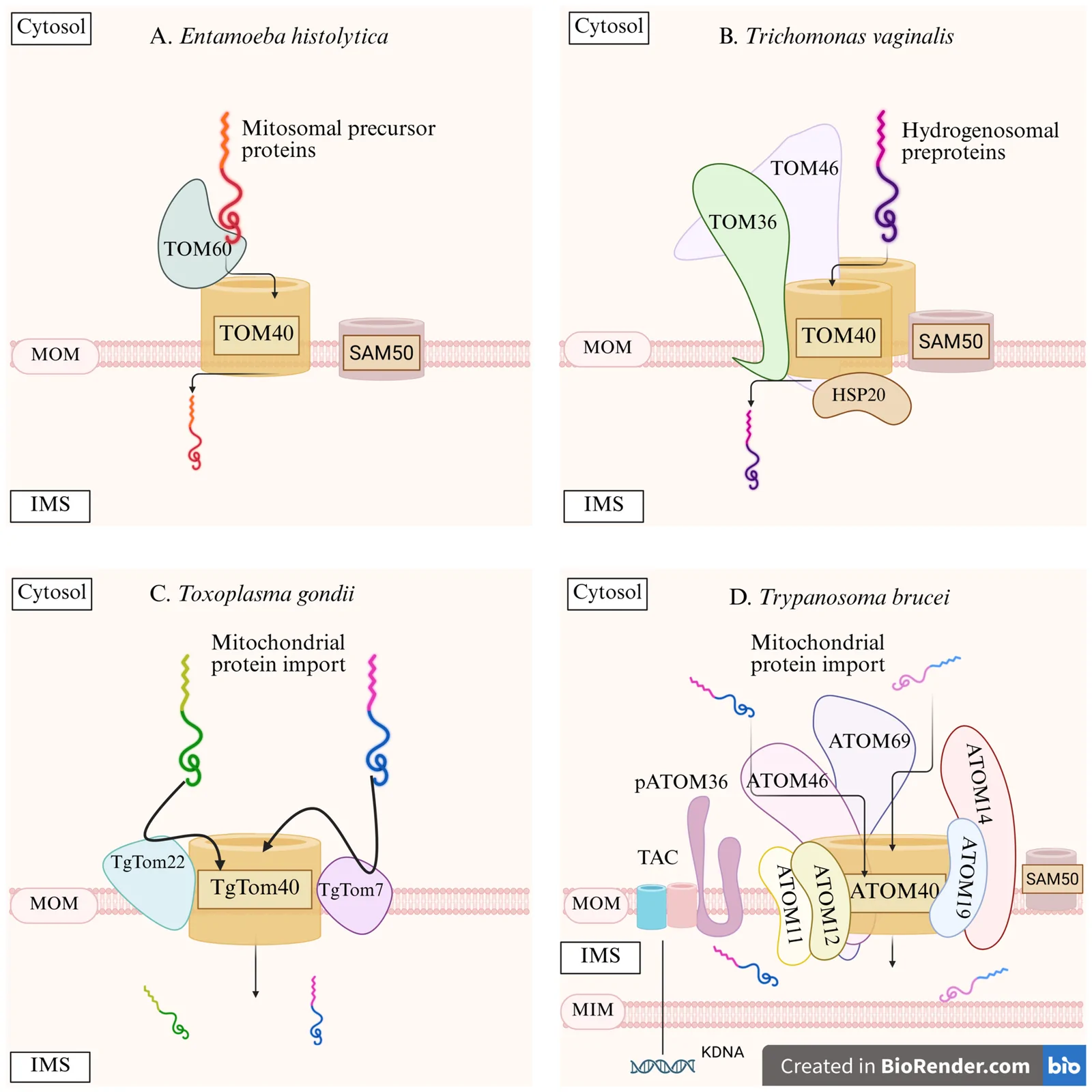
Evolutionary diversity and functional remodeling of the mitochondrial TOM complex across different parasites. The structural and functional variations in the TOM complex in distinct parasitic protists, highlighting lineage-specific adaptations in mitochondrial protein import machinery. (**A**) In *E. histolytica*, a unique receptor-like protein, Tom60, is proposed to recognize and guide mitosomal precursor proteins toward the central channel Tom40. (**B**) In *T. vaginalis*, protein translocation relies on a simplified Tom40-based channel assisted by accessory components Tom36 and Tom46, which cooperate with HSP20 to facilitate the import of hydrogenosomal preproteins through the Tom40 pore. (**C**) In *T. gondii*, the TOM machinery is more elaborate, where TgTom40 associates with accessory subunits TgTom22 and TgTom7, enhancing substrate recognition and import efficiency into the mitochondrial outer membrane. (**D**) In *T. brucei*, the ATOM complex exhibits the highest degree of diversification, where ATOM40 functions as the central pore, supported by multiple accessory proteins (ATOM11, ATOM12, ATOM19, ATOM46, and ATOM69) that collectively regulate protein docking and translocation. pATOM36 in *T. brucei* displays a dual function, contributing both to mitochondrial protein import and to the tripartite attachment complex (TAC), which physically links the mitochondrial genome to the basal body of the flagellum. Note that Tom40 and ATOM40 are not molecular homologs although both are functional analogues, forming protein-conducting channels. Created in BioRender. ka, K. (2026) https://BioRender.com/a5w3auf.

In *E. histolytica*, the mitosomal TOM complex lacks all known canonical cytosolic receptors, including Tom20 and Tom70. Alternatively, a novel protein termed Tom60 functions as the dedicated cytosolic receptor, containing tetratricopeptide repeat (TPR) domains that directly bind to the targeting signals of mitosomal precursor proteins (Figure 1A) [18,20]. This receptor represents a remarkable instance of the convergent evolution of a protein import factor, compensating for the loss of the canonical receptor system in this deeply divergent parasitic eukaryote.

### 3.2. T. vaginalis and Lineage-Specific Receptors

The hydrogenosomes of *T. vaginalis* possess a highly divergent TOM complex that incorporates lineage-specific, tail-anchored receptors—Tom36 and Tom46—which possess Hsp20 and TPR domains and can bind hydrogenosomal preproteins (Figure 1B) [21]. The absence of canonical receptors like Tom20 and Tom70, coupled with the presence of these novel, independently evolved subunits, underscores the remarkable plasticity of the protein import machinery in highly adapted parasitic organelles [21,22].

### 3.3. Highly Reduced State of Microsporidian Mitosomes

The microsporidian parasite *Encephalitozoon cuniculi* possesses mitosomes that represent a striking example of reductive evolution among mitochondria-related organelles [23]. These organelles have lost nearly all canonical mitochondrial functions, retaining only a minimal protein import apparatus essential for the import of a small number of proteins required for iron–sulfur cluster assembly. The TOM complex in *E. cuniculi* mitosomes is extraordinarily simplified, consisting of a Tom40 channel with only Tom70 retained as a receptor, while all other canonical receptors and accessory subunits have been lost [23]. This high reduction underscores the minimal requirements for mitochondrial protein import: a protein-conducting channel and at least one receptor to mediate precursor recognition. The retention of Tom70 rather than Tom20 in this system is notable and suggests that the substrate specificity of the import machinery is tailored to the limited set of proteins that must be imported into these highly reduced organelles [23].

## 4. The TOM Complex in Apicomplexan Parasites

### 4.1. A Divergent and Essential Import Machinery in Toxoplasma gondii

Characterization of the mitochondrial protein import machinery in *T. gondii* reveals a highly divergent and essential TOM complex. This minimalist translocon consists of a core protein-conducting channel formed by TgTom40—functionally conserved but phylogenetically distinct from its yeast and mammalian Tom40 homologs—alongside two essential accessory subunits: TgTom22, which acts as the central receptor and is critical for complex assembly, mitochondrial protein import, and parasite survival; and TgTom7, identified through a conserved motif-based search, which is integral to the stability of the ~400 kDa complex (Figure 1C). Notably, this streamlined apparatus lacks the accessory receptor subunits Tom20, Tom70, and Tom5 typical of model eukaryotes. Functional studies confirm that both TgTom22 and TgTom7 are not only essential for tachyzoite growth but are directly involved in mitochondrial protein import, underscoring a complex where conserved core functions are maintained through a lineage-specific, reduced architecture that highlights the adaptive evolution of this obligate intracellular parasite [8,24].

### 4.2. Integration with Organellar Function in Apicomplexans

The TOM complex in apicomplexans is integrated with broader mitochondrial functions through connections with various protein networks. A critical finding is the essential role of the coiled-coil-helix-coiled-coil-helix (CHCH) domain proteins CHCHD3 and CHCHD4 in the stability of the TOM complex in *T. gondii*. Genetic depletion of either protein resulted in a severe impairment of mitochondrial protein import, which was mechanistically linked to a significant loss of the central receptor subunit, TgTom22, and a concomitant reduction in other TOM complex components [25].

In *Plasmodium falciparum*, a recent transcriptomic study of clinical isolates associated with severe disease identified a co-expression network encompassing genes for various mitochondrial processes [26]. Within this network, the gene encoding a component of the TOM complex was differentially regulated, placing the mitochondrial import machinery within a broader transcriptional signature linked to parasite virulence and suggesting a potential connection between mitochondrial function and the pathogenesis of severe malaria [26].

## 5. The ATOM Complex: Convergent Evolution in Trypanosomatids

Trypanosomatids, including *Trypanosoma brucei* and *Leishmania* species, possess a fundamentally different mitochondrial outer membrane translocase termed the ATOM complex (Figure 1D). This complex represents a complete replacement of the canonical TOM, with its central pore formed not by a Tom40 ortholog but by ATOM40. Phylogenetic analyses place ATOM40 within the YtfM subgroup of the bacterial Omp85 superfamily, indicating a direct bacterial origin for this core import channel, making it a functional relic of an archaic protein transport system [10]. The receptor subunits are entirely different and represent a remarkable product of convergent evolution [7].

The primary import receptors in trypanosomatids are ATOM46 and ATOM69, which are functional analogues of Tom20 and Tom70, respectively, yet share no sequence or structural homology with their opisthokont counterparts. For instance, ATOM46 contains armadillo repeats instead of tetratricopeptide repeats (TPR) and exhibits an inverted membrane topology compared to yeast Tom20. Similarly, ATOM69, while containing TPR motifs like Tom70, has a C-terminal transmembrane domain (in contrast to Tom70’s N-terminal anchor) and contains an additional heat shock protein 20 (Hsp20) domain absent from Tom70. This fundamental divergence indicates that the protein import receptors were likely not present in the last eukaryotic common ancestor but evolved independently in the major eukaryotic supergroups; however, this inference remains subject to revision as additional lineages are characterized [7].

The ATOM complex core includes several additional essential, kinetoplastid-specific subunits. Functional and biochemical analyses have identified TbLOK1 as a novel core component, now designated ATOM19. Its depletion abolishes mitochondrial protein import, and it co-migrates in stable association with other ATOM components including ATOM40, ATOM46, ATOM14, ATOM12, and ATOM11, forming an integral structural network [27]. The specific functions of ATOM11, ATOM12, and ATOM14 are less characterized, but their stable association with the core pore suggests roles in complex assembly, stability, or substrate specificity. The ATOM complex is further regulated by pATOM36, an essential, novel, and trypanosomatid-specific integral outer membrane protein associated with the complex. It is required for the import of a specific subpopulation of nuclear-encoded matrix proteins, with dependency determined by the substrate’s N-terminal presequence domain [28]. pATOM36 is not a stoichiometric subunit of the mature translocase but is critical for its correct assembly; its ablation leads to the proteasomal degradation of several ATOM subunits. Interestingly, pATOM36 has a dual function, also participating in the tripartite attachment complex (TAC) that connects the mitochondrial genome to the basal body of the flagellum. A distinct fraction of pATOM36 localizes to the TAC, where it interacts with TAC65. RNAi depletion disrupts this link, causing severe kinetoplast DNA (kDNA) missegregation, thereby coupling mitochondrial biogenesis to genome inheritance [11]. The distinct and essential nature of these parasite-specific components, which have no direct homologues in mammalian cells, positions the ATOM complex as a potentially promising target for selective antiparasitic drugs [29,30]. However, direct evidence of selective inhibition has not yet been demonstrated, and the development of compounds capable of effectively targeting this membrane-embedded complex remains challenging.

A comparative overview of the mitochondrial protein import machinery across diverse parasitic protists is presented in Table 1. This table compares the composition and key features of the outer membrane translocase complexes across representative parasites with canonical mitochondria or mitochondrion-related organelles (MROs). Table 1 highlights the conservation of the core channel-forming component (Tom40/ATOM40) alongside remarkable lineage-specific variations in receptor subunits and accessory components.

## 6. Beyond Protein Import: Novel Roles and Integrated Functions

Mitochondrial protein import, mediated by the TOM or ATOM complexes, is a cornerstone of organellar biogenesis. However, in parasitic systems, this fundamental machinery frequently transcends its canonical role. It is co-opted into pathways central to the parasitic lifestyle, including direct manipulation of the host cell, surveillance of organellar homeostasis, and contributing to infection-associated pathophysiologies. This section explores these extended functions, highlighting the action of the import machinery as a critical nexus integrating mitochondrial function with parasite survival and virulence.

### 6.1. The TOM Complex in Host–Pathogen Interactions

Beyond its canonical role in mitochondrial biogenesis, the TOM complex has emerged as a critical platform in the innate immune response, making it a frequent target for viral and parasitic subversion. A key mechanism involves Tom70, which, in addition to its protein import functions, interacts with the mitochondrial antiviral signaling protein (MAVS) to form a signaling platform essential for initiating an interferon response [34,35]. This strategic role in immunity renders the TOM complex vulnerable to pathogen-derived effector proteins.

Proteomics has provided critical insights into host–pathogen interactions, to characterize infection-induced changes in the host cellular landscape [36,37,38,39]. One striking example of this subversion is observed with the intracellular parasite *T. gondii*. A pivotal study elucidated the central role of the host mitochondrial import receptor Tom70 in mediating the intimate association between *T. gondii* parasitophorous vacuoles and host mitochondria. Through quantitative proteomic analyses, the parasite mitochondria association factor 1B (MAF1b) was identified as specifically interacting with a select group of host mitochondrial proteins, with subsequent functional validation demonstrating that Tom70 and its associated chaperone HSPA9 are essential for this host mitochondrial association phenotype. Furthermore, MAF1b expression drives a significant enrichment and sequestration of host Tom70 at the parasitophorous vacuole membrane, highlighting a sophisticated mechanism by which an intracellular pathogen co-opts the host’s mitochondrial protein import machinery to reposition the organelle, thereby enhancing its intracellular fitness [40,41].

Recent research has further revealed that *T. gondii* infection triggers profound remodeling of the host mitochondrial outer membrane through the formation of structures termed structures positive for outer mitochondrial membrane (SPOTs). This process, which restricts mitochondrial fusion and facilitates parasite proliferation, is orchestrated by the parasite effector protein MAF1b (TgMAF1), which hijacks the host mitochondrial import receptor Tom70. Tom70 in turn recruits the host outer membrane insertase SAM50—the central complex responsible for assembling β-barrel proteins into the membrane—to initiate SPOT biogenesis, demonstrating that strategic hijacking of a single host import factor can drive extensive reorganization of host organellar structure [41,42].

### 6.2. Surveillance and Quality Control of Mitochondrial Import

The integrity of the mitochondrial protein import machinery is monitored by quality control pathways across diverse parasites. In *T. brucei*, a novel nuclear quality control pathway monitors the integrity of the mitochondrial protein import machinery [43]. When the import capacity of the TOM (ATOM) complex is compromised—either by genetic depletion of a key mitochondrial protease or by the mistargeting of aggregation-prone proteins—it triggers a retrograde signal to the nucleus. This signal initiates the ubiquitin-mediated degradation of a specific nuclear protein, leading to widespread downregulation of mRNAs encoding mitochondrial proteins. Thus, the TOM complex serves as the critical initial sensor where import failure activates a compensatory transcriptional response to alleviate proteotoxic stress and restore mitochondrial import homeostasis [43].

### 6.3. The TOM Complex as a Nexus in Infection-Associated Pathologies

**Figure 2:**
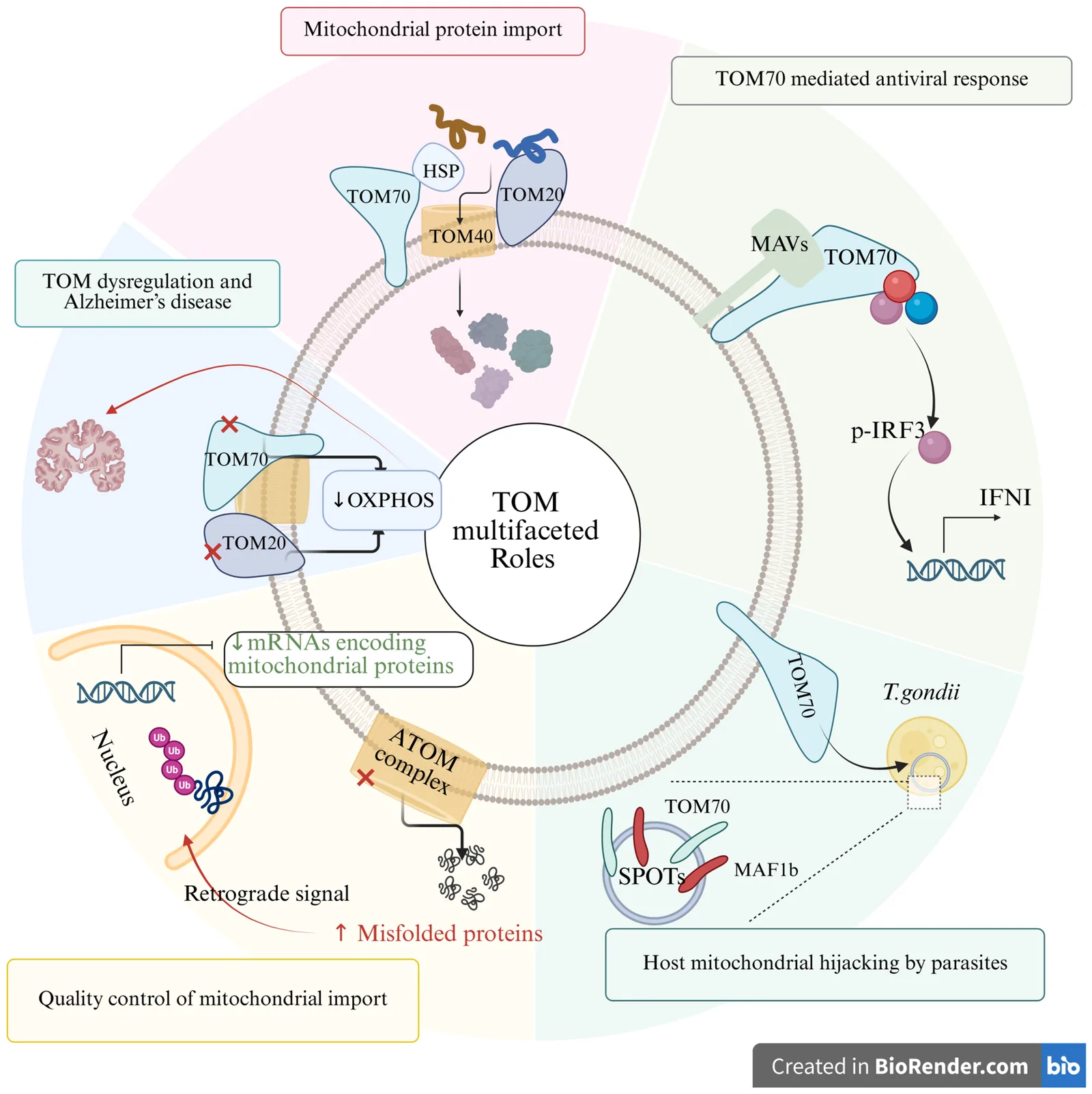
Multifunctional roles of the ATOM/TOM complex in mitochondrial biology and host interactions. Diverse functions of the ATOM/TOM complex beyond mitochondrial protein import through the Tom40 channel are summarized herein. In addition to its canonical role in protein translocation, Tom70 participates in antiviral signaling via interaction with MAVS, leading to activation of IRF1-mediated immune responses. Some parasites can hijack host Tom70, restricting mitochondrial fusion and promoting parasite proliferation. The complex also contributes to mitochondrial import surveillance and quality control by initiating retrograde signaling in response to misfolded protein accumulation, thereby modulating nuclear expression of mitochondrial proteins. Furthermore, dysfunction of TOM components (Tom70 and Tom20) has been associated with impaired oxidative phosphorylation and progression of neurodegenerative disorders such as Alzheimer’s disease. Created in BioRender. ka, K. (2026) https://BioRender.com/a5w3auf.

The TOM complex may represent a shared mechanistic node connecting the pathogenesis of clinically distinct diseases. The emerging epidemiological association between *T. gondii* infection and an increased risk for Alzheimer’s disease prompts the exploration of potential molecular synergies at the level of the TOM complex [44]. In the context of Alzheimer’s disease, the TOM complex shows marked dysfunction, evidenced by significant reductions in Tom70 and Tom20 that correlate directly with deficits in oxidative phosphorylation complexes [45]. Intriguingly, this mirrors the pathogen-induced subversion of the TOM complex during *T. gondii* infection, where the parasite effector MAF1b directly hijacks host Tom70 [41]. This convergence of pathological mechanisms on a key mitochondrial import receptor suggests a shared pathway to dysfunction, wherein the dysregulation of the TOM complex—whether through the progressive accumulation of neurodegenerative pathology or the acute, targeted manipulation by a persistent pathogen—represents a critical point of vulnerability for mitochondrial homeostasis. The diverse non-canonical functions of the TOM complex and related machinery in parasitic systems are summarized in Table 2 and illustrated in Figure 2. These functions include manipulation of host cell biology, coordination of organellar inheritance, surveillance of mitochondrial homeostasis, and participation in innate immune signaling (including the Tom70-mediated antiviral response shown in Figure 2). Together, these examples illustrate how the TOM/ATOM complex serves as a central hub integrating mitochondrial biogenesis with broader cellular and organismal processes.

## 7. Evolutionary Dynamics and Diversification of Mitochondrial Import Machinery

The comprehensive analysis of mitochondrial protein import systems across diverse parasitic protists reveals several fundamental evolutionary principles. First, the requirement for a protein-conducting channel at the outer membrane appears to be universal, maintained even in the most reduced MROs [31]. This functional conservation underscores the non-redundant role of the protein import gate, fundamental to the identity and function of the mitochondrion as an organelle. However, a critical conceptual distinction must be drawn between functional conservation and molecular homology in this context. While Tom40 (present in opisthokonts, plants, and most eukaryotes) and ATOM40 (found in trypanosomatids) perform the analogous function of forming the central protein-conducting pore, they are not orthologs. Tom40 belongs to the porin superfamily of β-barrel proteins, whereas ATOM40 is a member of the Omp85 superfamily, which is of direct bacterial origin [10]. Thus, while the function of a protein-conducting channel is highly conserved across all mitochondria and MROs, the molecular identity of this channel has been subject to substitution in the trypanosomatid lineage. This represents a case of non-homologous replacement rather than divergent evolution of a shared ancestral component. In contrast, the receptor systems that recognize cytosolic precursor proteins show remarkable plasticity, with multiple instantiations of convergent evolution leading to functionally analogous but structurally distinct receptors in different lineages [21].

The composition of the import machinery in parasites has been shaped by the functional constraints of their organelles, leading to distinct reductive and adaptive paths. Parasites with highly reduced MROs that have lost many mitochondrial functions typically possess similarly reduced import machinery, often lacking multiple receptor components [23]. However, the pattern of reduction differs among parasite lineages, which is consistent with convergent evolution driven by similar selective pressures rather than shared ancestry. For example, while *E. histolytica* has lost canonical receptors and evolved a novel Tom60, *E. cuniculi* has retained Tom70 but lost other receptors, and *T. vaginalis* has evolved entirely new receptor classes [21,23].

The discovery of a functional Tom70 homolog in the anaerobic mitochondrion-related organelle of the stramenopile parasite *Blastocystis* sp. [48] has significant implications for our understanding of the evolution of the mitochondrial import apparatus. This finding, confirmed through heterologous complementation in yeast, has led to the inference that Tom70 may have been present in the last eukaryotic common ancestor [48]. However, this remains an evolutionary inference based on phylogenetic distribution rather than a settled fact. An alternative interpretation—that Tom70 evolved independently in the stramenopile and opisthokont lineages through convergent evolution—cannot be definitively excluded without further structural and functional studies across a broader range of eukaryotic lineages. Consequently, the absence of Tom70 in other major lineages, such as plants and excavates, is now more plausibly explained by widespread secondary loss, underscoring a dynamic evolutionary history for this key import receptor [48].

The hypothesis that the last eukaryotic common ancestor (LECA) lacked the receptor systems observed in modern eukaryotes is supported by the absence of Tom20 and Tom70 homologs in several major lineages, but this inference remains tentative given the limited taxonomic sampling of deep-branching eukaryotes [7]. It is equally plausible that these receptors were present in LECA and subsequently lost in multiple lineages, as suggested by the discovery of Tom70 in *Blastocystis* [48].

The observation that parasites with MROs often possess reduced import machinery is consistent with the hypothesis that streamlined complexes may represent adaptations to relatively stable intracellular habitats [8]. However, this correlation does not establish causation, and the reduced complexity may equally reflect the overall reductive evolution of the organelles themselves rather than a specific adaptation to the host environment.

## 8. Adaptation to Parasitic Lifestyles and Functional Integration with Cellular Systems

The diversity of mitochondrial import machinery in parasites reflects specialized adaptations to their unique intracellular environments and metabolic requirements. In extracellular parasites, like African trypanosomes found in insect vectors and mammalian hosts, the elaborate ATOM complex with its unique receptor systems may represent an adaptation to the dramatically different environments encountered during their life cycle [7,17]. Conversely, in intracellular parasites like *T. gondii* and *Plasmodium* spp., the streamlined TOM complex may reflect the relatively stable intracellular niche these parasites inhabit [7,8]. While the presence of lineage-specific receptors appears to correlate with parasitic lifestyles, establishing a direct causal relationship between these innovations and survival in specific host environments requires further functional validation. These associations were therefore framed as hypotheses supported by correlative evidence rather than as established facts.

The reductive evolution observed in the import machinery of parasites with MROs mirrors the overall functional reduction in these organelles [17]. As metabolic pathways were lost during adaptation to anaerobic or intracellular environments, the requirement for importing the associated proteins disappeared, leading to the elimination of specialized import pathways or receptors. However, the retention of a minimal import apparatus, however divergent, highlights that protein import remains an essential function for all mitochondria and MROs, required at minimum for the insertion of the core Tom40 channel itself and the components of the retained essential pathways such as iron–sulfur cluster assembly.

Beyond its core role in protein import, the TOM complex in parasites is integrated with diverse cellular systems in ways that reflect their adapted biology. In *T. brucei*, the integration of the ATOM complex with the tripartite attachment complex (TAC) physically links mitochondrial protein import to the segregation of the single-unit mitochondrial genome and its connection to the flagellar basal body [30]. This unique configuration represents a fundamental adaptation to the complex single-unit mitochondrial genome of kinetoplastids and its precise segregation during cell division. The dual-role protein pATOM36 is a key physical and functional integrator in this system. While a fraction is associated with the ATOM complex to facilitate its assembly, a distinct pool localizes to the TAC where it interacts with component TAC65. Depletion of pATOM36 disrupts this link, leading to kinetoplast DNA missegregation and loss, thereby directly coupling the integrity of the protein import machinery to the faithful inheritance of mitochondrial DNA [11].

The emerging role of the host TOM complex as a target for parasitic manipulation represents another layer of functional integration. By targeting Tom70, *T. gondii* is able to reposition host mitochondria to its parasitophorous vacuole, potentially accessing nutrients or modulating host cell signaling pathways to promote intracellular survival. This subversion of the host import machinery underlines its strategic importance in cellular physiology and reveals how parasites can extend their influence beyond their own organelles to manipulate host cell functions [40].

## 9. Evidence Levels and Limitations

This review synthesizes findings from a diverse range of studies employing various experimental approaches, from genetic and biochemical characterizations to structural and bioinformatic analyses. However, several important limitations must be acknowledged when interpreting the evolutionary and functional conclusions presented herein.

First, the taxonomic sampling of parasitic protists remains incomplete. While we have covered the major lineages for which data are available—including kinetoplastids, apicomplexans, and several anaerobic protists—many parasitic lineages remain poorly characterized with respect to their mitochondrial import machinery. The conclusion that Tom40 is functionally conserved across all mitochondria and MROs is based on the organisms studied to date; it remains possible that additional lineages have replaced this channel with alternative systems, as observed with ATOM40 in trypanosomatids.

Second, the functional validation of components varies considerably across different systems. For some organisms (e.g., *T. brucei*, *T. gondii*), multiple components have been rigorously characterized through genetic depletion, biochemical reconstitution, and structural studies. For others (e.g., *E. cuniculi*, *Blastocystis*), the characterization relies more heavily on sequence-based predictions or limited functional assays. Table 1 indicates the level of evidence for each organism to provide transparency in this regard.

Third, many of the evolutionary inferences discussed—particularly those concerning the last eukaryotic common ancestor and the timing of gene losses or innovations—are based on phylogenetic analyses that are inherently limited by the available taxon sampling and the challenges of inferring deep evolutionary events. We have endeavored to present these inferences with appropriate caveats, distinguishing between established findings and hypotheses.

Fourth, the study of host–pathogen interactions involving the TOM complex is still in its early stages. The molecular details of how parasites manipulate host import machinery, and the functional consequences of these interactions, remain incompletely understood. Future studies based on approaches such as cryo-electron microscopy, in vivo imaging, and genetic manipulation of host–parasite systems will be essential to advance this field.

Finally, while the therapeutic potential of targeting parasite-specific import components is conceptually appealing, significant hurdles remain in translating these insights into clinical applications. The development of membrane-permeable inhibitors that selectively target protein–protein interactions or membrane-embedded channels may represent a promising challenge in medicinal chemistry.

## 10. Conclusions and Future Perspectives

This comprehensive synthesis of the mitochondrial protein import machinery in parasitic protists reveals both remarkable conservation of core principles and striking lineage-specific innovations. The universal functional requirement for a protein-conducting channel at the outer membrane—whether mediated by Tom40, ATOM40, or lineage-specific variants—underscores its fundamental role in defining mitochondrial identity, while the diverse array of receptor systems demonstrates the plasticity of evolutionary solutions to the challenge of substrate recognition. The functional adaptation of these import systems to specialized parasitic lifestyles—from the elaborate ATOM complex of trypanosomes to the minimalist systems of MROs—sheds light on how essential cellular machinery can be extensively modified through evolution.

Several promising directions for future research emerge from this synthesis. First, the structural characterization of divergent import complexes like the trypanosomal ATOM complex and the *Entamoeba* Tom60-containing complex would provide invaluable insights into the molecular mechanisms of convergent evolution in protein import. Second, the functional interrogation of novel lineage-specific components across diverse parasites will likely reveal new paradigms of protein recognition and membrane translocation. Third, the exploration of the strategies by which mitochondrial import interfaces with other cellular processes—such as stress responses, organelle dynamics, and quality control pathways—in parasitic systems may uncover novel regulatory mechanisms.

From a translational perspective, the significant divergence between parasite and host mitochondrial import machinery, particularly in trypanosomatids with their unique ATOM complex, may present an opportunity for therapeutic development that warrants further investigation [29]. The essential nature of these complexes for parasite survival, coupled with their structural distinction from host machinery, makes them attractive candidates for drug targeting studies. However, the development of selective inhibitors will require extensive medicinal chemistry and validation efforts. Similarly, understanding how parasites like *T. gondii* subvert the host TOM complex may inform strategies to block these interactions and limit infection.

Finally, the study of mitochondrial import in parasitic protists transcends its immediate context, providing a powerful paradigm for understanding eukaryotic cell evolution. These divergent systems function as natural experiments, delineating the universally essential core components of organellar biogenesis from the adaptable elements—like receptors and regulatory subunits—that can be innovated or discarded. The pervasive evolutionary plasticity observed—from receptor replacement to complex reconfiguration—underscores that essential cellular machinery is subject to intense selective pressures, yielding sophisticated and lineage-specific solutions. Research on these divergent and specialized parasites will remain indispensable for uncovering fundamental knowledge of membrane biology, organellar evolution, and the dynamic interplay between endosymbiotic organelles and their host cells.

## Figures and Tables

**Table 1:** Diversity of mitochondrial protein import machinery in selected parasitic protists.

Parasite	Organelle Type	Core Channel	Key Accessory/Receptor Components	Unique Features	Evidence Level	Reference
*Trypanosoma brucei*	Mitochondrion	ATOM40	ATOM46, ATOM69, ATOM19, ATOM14, ATOM12, ATOM11; pATOM36 (assembly regulator)	Bacterial-origin pore (Omp85 superfamily); unique receptor subunits; dual-function pATOM36 links import to kDNA inheritance	Demonstrated	[7,10,11,27,28]
*Toxoplasma gondii*	Mitochondrion	TgTom40	TgTom22, TgTom7	Minimalist complex; lacks Tom20/Tom70; essential for tachyzoite growth	Demonstrated	[8,24]
*Entamoeba histolytica*	Mitosome	Tom40	Tom60 (novel receptor), MBOMP30	Novel Tom60 receptor with TPR domains; loss of canonical receptors.	Supported by interaction studies	[18,20,31]
*Giardia intestinalis*	Mitosome	GiTom40	GIMiT partner protein	Highly reduced; novel interactors identified	Supported by interaction studies	[32,33]
*Trichomonas vaginalis*	Hydrogenosome	TvTom40-2	Tom36, Tom46 (lineage-specific)	Lineage-specific TA-protein receptors with Hsp20/TPR domains	Demonstrated	[21,22]
*Encephalitozoon cuniculi*	Mitosome	Tom40	Tom70 (only)	Extremely reduced; only Tom70 retained as a receptor	Predicted	[23]

Abbreviations: MRO, mitochondrion-related organelle; TA-protein, tail-anchored protein; TPR, tetratricopeptide repeat; kDNA, kinetoplast DNA. Footnote: Tom40 and ATOM40 are functional analogues (both forming protein-conducting channels) but are not molecular homologs; Tom40 belongs to the porin superfamily, whereas ATOM40 belongs to the Omp85 superfamily of bacterial origin.

**Table 2:** Non-import functions of TOM/ATOM complexes in parasite and host systems.

**Function**	**Parasite/System**	**Key Components**	**Biological Role**	**Evidence Level**	**Reference**
Host mitochondrial association	*Toxoplasma gondii*	Host Tom70, HSPA9, MAF1b	Recruitment of host mitochondria to parasitophorous vacuole	Demonstrated	[41]
Mitochondrial outer membrane remodeling	*Toxoplasma gondii*	Host Tom70, SAM50, MAF1b	Formation of SPOT; restriction of mitochondrial fusion	Demonstrated	[41]
Genome segregation	*Trypanosoma brucei*	pATOM36, TAC	Links mitochondrial DNA to basal body of flagellum	Demonstrated	[7]
Import surveillance	*Trypanosoma brucei*	ATOM complex	Activates nuclear quality control upon import failure	Supported by interaction studies	[43]
Antiviral innate immune signaling	Mammalian (host) cells	Host Tom70, MAVS	Platform for Type I interferon (IFN-I) response; phosphorylation of IRF3; targeted by pathogens	Demonstrated	[46,47]

Abbreviations: IFN-I, Type I interferon; IRF3, interferon regulatory factor 3; kDNA, kinetoplast DNA; MAVS, mitochondrial antiviral signaling protein; SPOTs, Structures Positive for Outer Mitochondrial Membrane; TAC, tripartite attachment complex.

## Data Availability

No new data were created or analyzed in this study. Data sharing is not applicable to this article.

## References

[1] Araiso Y., Endo T. (2022). Structural overview of the translocase of the mitochondrial outer membrane complex. Biophys. Physicobiol..

[2] Nussberger S., Ghosh R., Wang S. (2024). New insights into the structure and dynamics of the TOM complex in mitochondria. Biochem. Soc. Trans..

[3] Sherman E.L., Go N.E., Nargang F.E. (2005). Functions of the small proteins in the TOM complex of *Neurospora crasssa*. Mol. Biol. Cell.

[4] Guan Z., Yan L., Wang Q., Qi L., Hong S., Gong Z., Yan C., Yin P. (2021). Structural insights into assembly of human mitochondrial translocase TOM complex. Cell Discov..

[5] de Paula W.B., Allen J.F., van der Giezen M. (2011). Mitochondria, hydrogenosomes and mitosomes in relation to the CoRR hypothesis for genome function and evolution. Organelle Genetics: Evolution of Organelle Genomes and Gene Expression.

[6] Mogi T., Kita K. (2010). Diversity in mitochondrial metabolic pathways in parasitic protists Plasmodium and Cryptosporidium. Parasitol. Int..

[7] Schneider A. (2018). Mitochondrial protein import in trypanosomatids: Variations on a theme or fundamentally different?. PLoS Pathog..

[8] Van Dooren G.G., Yeoh L.M., Striepen B., McFadden G.I. (2016). The Import of Proteins into the Mitochondrion of *Toxoplasma gondii*. J. Biol. Chem..

[9] Van Der Giezen M. (2009). Hydrogenosomes and mitosomes: Conservation and evolution of functions 1. J. Eukaryot. Microbiol..

[10] Pusnik M., Schmidt O., Perry A.J., Oeljeklaus S., Niemann M., Warscheid B., Lithgow T., Meisinger C., Schneider A. (2011). Mitochondrial preprotein translocase of trypanosomatids has a bacterial origin. Curr. Biol..

[11] Käser S., Oeljeklaus S., Týč J., Vaughan S., Warscheid B., Schneider A. (2016). Outer membrane protein functions as integrator of protein import and DNA inheritance in mitochondria. Proc. Natl. Acad. Sci. USA.

[12] Wang W., Chen X., Zhang L., Yi J., Ma Q., Yin J., Zhuo W., Gu J., Yang M. (2020). Atomic structure of human TOM core complex. Cell Discov..

[13] Jackson J., Becker T. (2025). Unclogging of the TOM complex under import stress. Biol. Chem..

[14] Saitoh T., Igura M., Obita T., Ose T., Kojima R., Maenaka K., Endo T., Kohda D. (2007). Tom20 recognizes mitochondrial presequences through dynamic equilibrium among multiple bound states. EMBO J..

[15] Sayyed U.M.H., Mahalakshmi R. (2022). Mitochondrial protein translocation machinery: From TOM structural biogenesis to functional regulation. J. Biol. Chem..

[16] Rampelt H., Sucec I., Bersch B., Horten P., Perschil I., Martinou J.-C., van Der Laan M., Wiedemann N., Schanda P., Pfanner N. (2020). The mitochondrial carrier pathway transports non-canonical substrates with an odd number of transmembrane segments. BMC Biol..

[17] Makiuchi T., Nozaki T. (2014). Highly divergent mitochondrion-related organelles in anaerobic parasitic protozoa. Biochimie.

[18] Santos H.J., Nozaki T. (2022). The mitosome of the anaerobic parasitic protist *Entamoeba histolytica*: A peculiar and minimalist mitochondrion-related organelle. J. Eukaryot. Microbiol..

[19] Leger M.M., Kolisko M., Kamikawa R., Stairs C.W., Kume K., Čepička I., Silberman J.D., Andersson J.O., Xu F., Yabuki A. (2017). Organelles that illuminate the origins of Trichomonas hydrogenosomes and Giardia mitosomes. Nat. Ecol. Evol..

[20] Makiuchi T., Mi-Ichi F., Nakada-Tsukui K., Nozaki T. (2013). Novel TPR-containing subunit of TOM complex functions as cytosolic receptor for Entamoeba mitosomal transport. Sci. Rep..

[21] Makki A., Rada P., Žárský V., Kereïche S., Kováčik L., Novotný M., Jores T., Rapaport D., Tachezy J. (2019). Triplet-pore structure of a highly divergent TOM complex of hydrogenosomes in *Trichomonas vaginalis*. PLoS Biol..

[22] Rada P., Doležal P., Jedelský P.L., Bursac D., Perry A.J., Šedinová M., Smíšková K., Novotný M., Beltrán N.C., Hrdý I. (2011). The core components of organelle biogenesis and membrane transport in the hydrogenosomes of Trichomonas vaginalis. PLoS ONE.

[23] Waller R.F., Jabbour C., Chan N.C., Celik N., Likic V.A., Mulhern T.D., Lithgow T. (2009). Evidence of a reduced and modified mitochondrial protein import apparatus in microsporidian mitosomes. Eukaryot. Cell.

[24] Usey M.M., Huet D. (2022). Parasite powerhouse: A review of the Toxoplasma gondii mitochondrion. J. Eukaryot. Microbiol..

[25] Usey M.M., Huet D. (2023). ATP synthase-associated coiled-coil-helix-coiled-coil-helix (CHCH) domain-containing proteins are critical for mitochondrial function in *Toxoplasma gondii*. mBio.

[26] Gujarati S., Singal B.R., Gupta D., Kochar S.K., Kochar D.K., Das A. (2026). Differential expression of mitochondria-associated genes in clinical samples of *Plasmodium falciparum* showing severe manifestations. BMC Genom..

[27] Desy S., Mani J., Harsman A., Käser S., Schneider A. (2016). TbLOK1/ATOM19 is a novel subunit of the noncanonical mitochondrial outer membrane protein translocase of *Trypanosoma brucei*. Mol. Microbiol..

[28] Pusnik M., Mani J., Schmidt O., Niemann M., Oeljeklaus S., Schnarwiler F., Warscheid B., Lithgow T., Meisinger C., Schneider A. (2012). An essential novel component of the noncanonical mitochondrial outer membrane protein import system of trypanosomatids. Mol. Biol. Cell.

[29] Pedra-Rezende Y., Bombaça A.C.S., Menna-Barreto R.F.S. (2022). Is the mitochondrion a promising drug target in trypanosomatids?. Mem. Inst. Oswaldo Cruz.

[30] Schimanski B., Aeschlimann S., Stettler P., Käser S., Gomez-Fabra Gala M., Bender J., Warscheid B., Vögtle F.-N., Schneider A. (2022). P166 links membrane and intramitochondrial modules of the trypanosomal tripartite attachment complex. PLoS Pathog..

[31] Santos H.J., Imai K., Makiuchi T., Tomii K., Horton P., Nozawa A., Ibrahim M., Tozawa Y., Nozaki T. (2015). A novel mitosomal β-barrel outer membrane protein in Entamoeba. Sci. Rep..

[32] Rout S., Zumthor J.P., Schraner E.M., Faso C., Hehl A.B. (2016). An interactome-centered protein discovery approach reveals novel components involved in mitosome function and homeostasis in Giardia lamblia. PLoS Pathog..

[33] Dagley M.J., Dolezal P., Likić V.A., Smid O., Purcell A.W., Buchanan S.K., Tachezy J., Lithgow T. (2009). The protein import channel in the outer mitosomal membrane of Giardia intestinalis. Mol. Biol. Evol..

[34] Kreimendahl S., Rassow J. (2020). The mitochondrial outer membrane protein Tom70-mediator in protein traffic, membrane contact sites and innate immunity. Int. J. Mol. Sci..

[35] den Brave F., Pfanner N., Becker T. (2024). Mitochondrial entry gate as regulatory hub. Biochim. Biophys. Acta (BBA)—Mol. Cell Res..

[36] Rashidi S., Mojtahedi Z., Shahriari B., Kalantar K., Ghalamfarsa G., Mohebali M., Hatam G. (2019). An immunoproteomic approach to identifying immunoreactive proteins in *Leishmania infantum* amastigotes using sera of dogs infected with canine visceral leishmaniasis. Pathog. Glob. Health.

[37] Rashidi S., Kalantar K., Hatam G. (2018). Using proteomics as a powerful tool to develop a vaccine against Mediterranean visceral leishmaniasis. J. Parasit. Dis..

[38] Dousti M., Manzano-Roman R., Rashidi S., Barzegar G., Ahmadpour N.B., Mohammadi A., Hatam G. (2021). A proteomic glimpse into the effect of antimalarial drugs on *Plasmodium falciparum* proteome towards highlighting possible therapeutic targets. Pathog. Dis..

[39] Rashidi S., Sanchez-Montejo J., Mansouri R., Ali-Hassanzadeh M., Savardashtaki A., Bahreini M.S., Karimazar M., Manzano-Román R., Nguewa P. (2022). Mining the proteome of Toxoplasma parasites seeking vaccine and diagnostic candidates. Animals.

[40] Blank M.L., Xia J., Morcos M.M., Sun M., Cantrell P.S., Liu Y., Zeng X., Powell C.J., Yates N., Boulanger M.J. (2021). *Toxoplasma gondii* association with host mitochondria requires key mitochondrial protein import machinery. Proc. Natl. Acad. Sci. USA.

[41] Li X., Straub J., Medeiros T.C., Mehra C., den Brave F., Peker E., Atanassov I., Stillger K., Michaelis J.B., Burbridge E. (2022). Mitochondria shed their outer membrane in response to infection-induced stress. Science.

[42] Schuler M.-H., Hughes A.L. (2022). SPOTting stress at the mitochondrial outer membrane. Mol. Cell.

[43] Dewar C.E., Oeljeklaus S., Mani J., Mühlhäuser W.W., von Känel C., Zimmermann J., Ochsenreiter T., Warscheid B., Schneider A. (2022). Mistargeting of aggregation prone mitochondrial proteins activates a nucleus-mediated posttranscriptional quality control pathway in trypanosomes. Nat. Commun..

[44] Chegeni T.N., Sarvi S., Moosazadeh M., Sharif M., Aghayan S.A., Amouei A., Hosseininejad Z., Daryani A. (2019). Is *Toxoplasma gondii* a potential risk factor for Alzheimer’s disease? A systematic review and meta-analysis. Microb. Pathog..

[45] Chai Y.L., Xing H., Chong J.R., Francis P.T., Ballard C.G., Chen C.P., Lai M.K. (2017). Mitochondrial translocase of the outer membrane alterations may underlie dysfunctional oxidative phosphorylation in Alzheimer’s disease. J. Alzheimer’s Dis..

[46] Lin R., Paz S., Hiscott J. (2010). Tom70 imports antiviral immunity to the mitochondria. Cell Res..

[47] Hurła M., Pikor D., Banaszek-Hurła N., Drelichowska A., Dorszewska J., Kozubski W., Kacprzak E., Paul M. (2025). Unraveling the Role of Proteinopathies in Parasitic Infections. Biomedicines.

[48] Tsaousis A.D., Gaston D., Stechmann A., Walker P.B., Lithgow T., Roger A.J. (2011). A functional Tom70 in the human parasite *Blastocystis* sp.: Implications for the evolution of the mitochondrial import apparatus. Mol. Biol. Evol..

